# The Rise of (Chiral) 3D Mechanical Metamaterials

**DOI:** 10.3390/ma12213527

**Published:** 2019-10-28

**Authors:** Janet Reinbold, Tobias Frenzel, Alexander Münchinger, Martin Wegener

**Affiliations:** 1 Institute of Applied Physics, Karlsruhe Institute of Technology, 76128 Karlsruhe, Germany; janetreinbold@gmail.com (J.R.); Tobias.Frenzel@kit.edu (T.F.); alexander.muenchinger@kit.edu (A.M.); 2 Institute of Nanotechnology, Karlsruhe Institute of Technology, 76021 Karlsruhe, Germany

**Keywords:** mechanical metamaterials, chirality, acoustical activity

## Abstract

On the occasion of this special issue, we start by briefly outlining some of the history and future perspectives of the field of 3D metamaterials in general and 3D mechanical metamaterials in particular. Next, in the spirit of a specific example, we present our original numerical as well as experimental results on the phenomenon of acoustical activity, the mechanical counterpart of optical activity. We consider a three-dimensional chiral cubic mechanical metamaterial architecture that is different from the one that we have investigated in recent early experiments. We find even larger linear-polarization rotation angles per metamaterial crystal lattice constant than previously and a slower decrease of the effects towards the bulk limit.

## 1. Introduction

**Figure 1:**
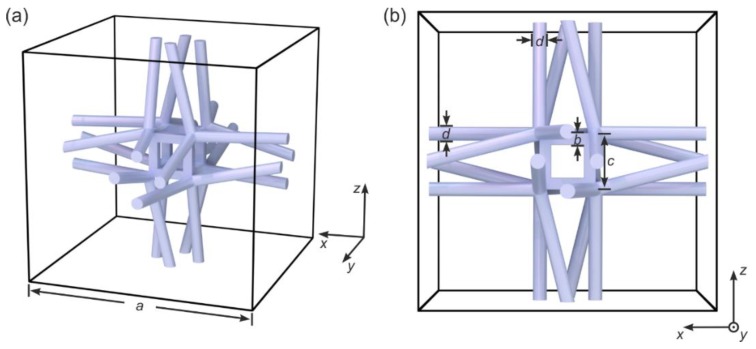
(**a**) and (**b**) are different views onto the blueprint of one cubic unit cell of the 3D chiral mechanical metamaterial lattice considered here. The indicated geometrical parameters are: a=250 µm, b=10 µm, c=50 µm, and d=6.25 µm. Parameters of the constituent polymer material are: Young’s modulus (or storage modulus) E=4.18 GPa, Poisson’s ratio ν=0.4, and mass density $\rho =1.15\;g/{\mathrm{cm}}^{3}$ (cf. [21]). For some of the calculations, we have added an imaginary part (or loss modulus) of 0.20 GPa to the quoted real part of the polymer Young’s modulus. Whenever applicable, we will explicitly mention this finite imaginary part, which describes damping of the elastic waves.

Consider the three-dimensional (3D) micro-lattice shown in Figure 1 as an example. When giving talks to a broad audience, an ever-reoccurring question is whether one should see such an artificial crystal as a “structure” or as a “material”. The true answer is that both viewpoints are permissible. However, treating such rationally designed lattices as material or “metamaterial” has the advantage that the properties of the lattice can be mapped onto simple effective-medium parameters, such as, for example, the effective Young’s modulus in mechanics. Working further with these effective-medium parameters to design systems eases the treatment compared to looking at an entire system as a micro- or nanostructure. By analogy, computer chips are successfully designed by using electric conductivities etc., whereas the design would likely be impossible if the entire computer chip needed to be treated on the level of individual atoms.

The “meta” in metamaterials emphasizes that these effective-medium properties go beyond the properties of the ingredient materials, qualitatively or quantitatively [1]. Sometimes, they can even go beyond what nature has to offer. However, one should be cautious when referring to nature. Nature offers a multitude of amazing microstructured materials, ranging from 3D photonic crystals in butterflies, beagles, and plants to mechanical materials such as wood, bone, or skin.

It should be said that the understanding of the notion of “metamaterials” has changed significantly over the last 20 years [1]. Originally, metamaterials were solely associated with man-made lattices with centimeter-scale periods leading to effective negative refractive indices in electromagnetism at GHz frequencies. In fact, early definitions explicitly restricted metamaterials to electromagnetic waves [1]. Miniaturization of the unit cells by orders of magnitude brought the field to optical and even to visible frequencies [2]. 

This ground sometimes made the publication of early work on mechanical metamaterials difficult. One of us distinctly remembers reviewers’ comments such as “… the authors misuse the term metamaterials …”, “… metamaterials require multiple different components. The structure discussed here has only one …”, “… metamaterials must address waves, whereas the authors speak about static properties …”, “… the static case lacks a characteristic length scale …” or “… the idea of mechanical metamaterials is not new. Many examples have been published decades ago …” etc. We know from personal discussions that others in the field of mechanical metamaterials faced very similar negative reactions early on. To some extent, these reactions were probably based on the fact that the notion “metamaterial” was a cash cow for funding in the early years of this century after the notion had been coined. For some, the idea of metamaterials just somehow sounded interesting and fashionable.

Having said that, we should humbly admit that the idea of artificial composite materials with unusual properties is not new. The 2002 textbook by Graeme W. Milton “The Theory of Composites” [3] summarizes the history as well as the underlying physics and mathematics on more than 700 pages. The word “metamaterial” does not appear once in this book though. In mechanics, for example, Roderik Lakes published early work on auxetics [4]. Milton and Cherkaev introduced the concept of pentamodes (or “anti-auxetics”) [5], from which any linearly elastic material describable by Cauchy elasticity can be constructed conceptually. However, experimental advances in nanofabrication and in 3D printing on the macro- and on the micro-scale have spurred interest tremendously and have in turn stimulated novel designs and design approaches [1]. 

Mechanical metamaterials offer opportunities yet beyond those of optical metamaterials. In the linear regime, mechanics is richer than optics. This statement has been proven mathematically [6] by arranging the equations of mechanics and electromagnetism into the same form. Intuitively, mechanics always comprises transverse and longitudinal excitations at the same time. In contrast, longitudinal waves can appear in optics in only a few rare exceptions. In the nonlinear mechanical regime, nonlinear geometric effects can lead to huge nonlinearities and multi-stable behavior even if the constituents behave purely linearly elastic [7,8]. In contrast, nonlinear effects in optics are almost always very small corrections of the linear properties. Recent reviews on mechanical metamaterials in general can also be found in [9,10,11].

Nevertheless, some effects that have been known for decades in optics have not been observed in mechanics until recently. Ultrasound acoustical activity emphasized in this paper is one such example.

More broadly, some of us have speculated previously [1] that the futures of 3D metamaterials and 3D printing may be closely linked: In 2D graphical printing (ink-jet or laser printing), one routinely uses the concept of “dithering” to generate thousands of apparent or effective colors from just four ink cartridges (black, cyan, magenta, and yellow). Looking through a microscope, one notices that the 2D printer can actually only print dot patterns, with each dot originating from one of these four cartridges. From a distance, the human eye cannot resolve this substructure anymore and the patterns appear as different homogeneous effective colors. The analogue of the “dithering” substructure in 3D material printing is the metamaterial unit cell. By tailoring the unit cell structure, thousands of different effective material properties can be achieved from just a few material cartridges of the 3D printer. The 3D micro-architectures discussed below are even composed of only a single polymer material and voids within.

## 2. Enhanced Acoustical Activity

The architecture shown in Figure 1 has been inspired by the one introduced in [12]. It is composed of helical wire bundles, each bundle with four individual wires or beams. The different bundles are connected via small cubic elements and are arranged on a simple-cubic translational lattice, leading to a 3D cubic chiral mechanical metamaterial. The constituent material (gray) shall be describable by ordinary linear Cauchy elasticity [13]. Chiral objects are distinct from their mirror image, hence chiral structures lack centrosymmetry, mirror symmetries, and rotation-reflection symmetries [14,15,16]. However, any effect of chirality of the mechanical metamaterial goes beyond the regime of Cauchy elasticity. In other words, chirality plays strictly no role in a Cauchy continuum [13]. In sharp contrast, Eringen micropolar elasticity [17,18] (sometimes also loosely referred to as Cosserat elasticity) and Willis elasticity [19] can describe effects of chirality and have led to good agreement with previous experimental results on 3D mechanical metamaterials [18] in the static regime. Various other chiral mechanical lattices have recently also been investigated in the static regime [18,20].

Here, we rather focus on the dynamic or wave regime of chiral architectures. The phenomenon of “acoustical activity” (alternatively named mechanical activity or elastic activity)—the mechanical counterpart of optical activity—was predicted theoretically years ago [22]. The notion “activity” stands for the rotation of the linear polarization axis of a transversely polarized optical or mechanical wave, with the rotation angle being proportional to the propagation distance and independent of the orientation of the incident linear polarization. To avoid confusion, we emphasize that the material itself is passive, meaning that neither external energy sources nor switchable material parameters are required. This rotation due to acoustical activity must not be confused with Faraday rotation and is due to the fact that the eigenmodes correspond to left-handed and right-handed circular polarization and that these eigenmodes propagate with different phase velocities. Following the original theoretical prediction [22], direct experiments on quartz crystals at about 1 GHz frequency were published [23]. Quartz is also a paradigm crystal for obtaining optical activity. Later, different continuum descriptions of acoustical activity were compared [24,25], all for the case of infinitely extended, that is bulk, crystals. 

More recently, we have presented an early experimental demonstration of acoustical activity in 3D mechanical metamaterials [21]. The effects observed here [21] have been much larger than the effects for ordinary crystals [23]. More importantly, one cannot change the optimum operation frequency for ordinary crystals, whereas it can easily be tailored by choice of the lattice constant in the case of 3D crystalline metamaterials. The aim of the present paper is to add a second experimental demonstration on a different chiral metamaterial lattice (cf. Figure 1). 

**Figure 2:**
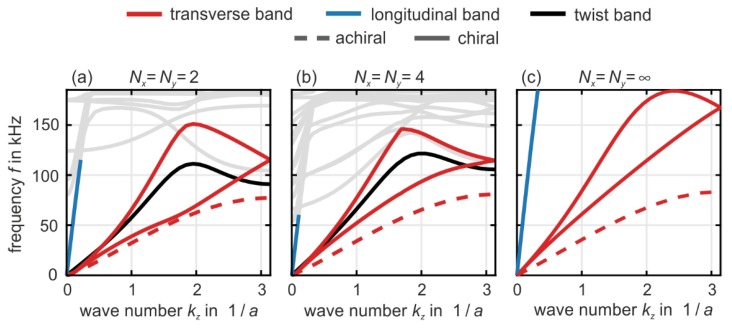
Calculated phonon band structures of metamaterial beams with a cross section of $N_{x}\times N_{y}$ unit cells in the xy-plane and with $N_{z}=\infty$ for wave vectors, $\vec{k}$, along the z -direction, i.e., $\vec{k}=\left(0,0,k_{z}\right)$ with wave number $k_{z}$. (**a**) $N_{x}=N_{y}=2$, (**b**) $N_{x}=N_{y}=4$, and (**c**) bulk with $N_{x}=N_{y}=\infty$. The transverse (or shear or flexural) bands are highlighted in red. Without chirality, the two transverse bands would be degenerate due to the four-fold rotational symmetry of the metamaterial crystal (see dashed red curves). The blue bands correspond to longitudinal-like (or pressure-like) and the black bands to twist-like modes, respectively. The higher bands are not important in the context of this paper and are plotted in light gray for clarity. Parameters have been given in Figure 1; the imaginary part of the polymer Young’s modulus is set to zero. In panel (a), the maximum splitting of the red bands, $\Delta k_{z}$, is about half of π/a, corresponding to a rotation angle of about 45° per lattice constant, which approach the fundamental bound of 90° per lattice constant.

In Figure 2, we show numerically calculated phonon band structures (i.e., eigenfrequencies versus wave number) of metamaterial beams based on the crystal unit cell depicted in Figure 1. The geometrical and polymer material parameters are given in the caption of Figure 1. The calculations in Figure 2 refer to metamaterial beams with a cross section of $N_{x}\;\times \;N_{y}$ unit cells (as indicated). The metamaterial beams are infinitely extended along the propagation direction of the waves (z-direction, wave number $k_{z}$). These solutions have been obtained by using the eigenmode solver MUMPS in the software package Comsol and by using Bloch-periodic boundary conditions along the z-direction. 

For finite values of $N_{x}=N_{y}$, we use traction-free boundary conditions at the metamaterial beam surface along the x- and the y-direction.

For $N_{x}=N_{y}=\infty$ (bulk case), we also use Bloch-periodic boundary conditions along the x- and the y-direction. This overall procedure is the same as in [21], where it was used for a different metamaterial architecture.

To ease the discussion, we have colored the bands in Figure 2 (see legend). However, this coloration has to be taken with a grain of salt because most bands are mixed in character. For example, in the presence of chirality, the longitudinal pressure bands (blue) and the twist bands (black) are mixed. This mixture is the immediate dynamic counterpart of the (quasi-)static push-to-twist conversion [18] (also see [21]). Furthermore, the transverse polarized bands or shear modes or TA phonons (red), which are strictly speaking flexural modes for beams with finite cross section, exhibit avoided crossings with localized (“optical”) modes at higher frequencies in the middle of the first Brillouin zone around $k_{z}\approx \frac{1}{2}\frac{\pi }{a}$. These higher-frequency bands are plotted in light gray because they are of lesser importance in the context of this article. The dashed red curve shows the two degenerate transverse bands in the achiral reference case. Here, each wire in the bundles of four (cf. Figure 1) is parallel to a principal cubic axis, i.e., we have eliminated the twisting of the bundles (not depicted). By comparison with the red chiral case in Figure 2, we see that chirality leads to a significant stiffening of the structure, thus to an increase of the phase velocity of the transverse bands.

The two red bands or chiral phonons [21] in Figure 2 are important in the context of acoustical activity. By the four-fold rotational symmetry along a principal cubic axis (hence also the z-axis), these two bands are degenerate in absence of chirality (dashed red curves). In the presence of chirality, at a given fixed angular frequency ω, the splitting of these two bands in phonon wave number, $\Delta k_{z}\left(\omega \right)$, determines the polarization-rotation angle, φ, via the equation [21]

(1) $$\varphi \left(\omega \right)=\Delta k_{z}\left(\omega \right)\frac{L_{z}}{2}$$

Here, $L_{z}\;\geq \;0$ is the propagation distance or length, and the factor $\Delta k_{z}\left(\omega \right)$ stems from the different phase velocities of left-handed and right-handed circular polarization (see above) [21]. The modulus of the rotation angle is fundamentally bounded by

(2) $$\left|\varphi \left(\omega \right)\right|\leq \frac{\pi }{2}\;\frac{L_{z}}{a}$$

that is, it cannot be larger than 90° per lattice constant a. This bound can be understood as follows. The two red bands correspond to circular polarization, which require three- or fourfold rotational symmetry along the axis of the wave vector $\vec{k}=\left(0,0,k_{z}\right)$. As usual, the two wave numbers $k_{z}=\pm \;\pi /a$ at the edge of the first Brillouin zone are equivalent. When replacing $k_{z}\;\to \;-k_{z}$, a left-handed circular mode turns into a right-handed and vice versa. Therefore, the left- and the right-handed mode must be degenerate at the Brillouin-zone edge. Thus, the maximum possible wave number difference for a propagating wave in either the positive or the negative $k_{z}$-direction at given frequency ω is $\left|\Delta k_{z}\left(\omega \right)\right|=\pi /a$. From here, the above bound follows immediately.

By comparison of Figure 2 with the band structures of another previously discussed 3D metamaterial [21], we find that the anticipated rotation angles per lattice constant are larger in the present paper by about a factor of two for a given value of $N_{x}=N_{y}$. The difference is largest for the bulk case of $N_{x}=N_{y}=N_{z}=\infty$. This aspect is beneficial for potential applications, in which one may want to convert one linear polarization into the orthogonal one (i.e., 90-degrees rotation) over a propagation distance as short as possible.

**Figure 3:**
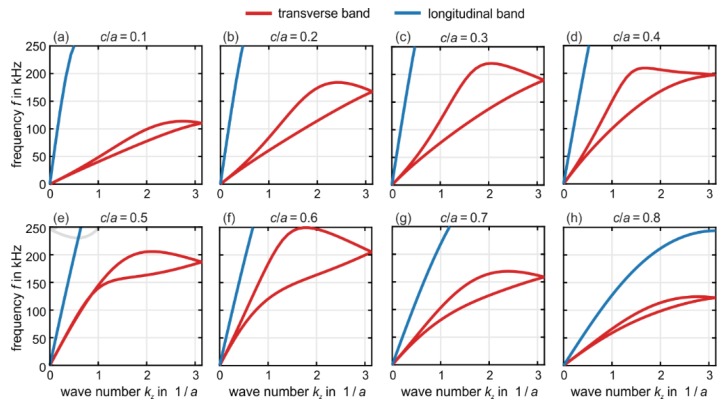
Calculated bulk phonon band structures (i.e., $N_{x}=N_{y}=N_{z}\;\to \;\infty )$ for the metamaterial defined in Figure 1. The lattice constant a and the other parameters are the same as in Figure 2c, where c/a=0.2, but we vary the c/a ratio (as indicated). The bands are colored as in Figure 2. In particular, the chiral transverse acoustical bands are again highlighted in red. (**a**) c/a=0.1, (**b**) c/a=0.2, (**c**) c/a=0.3, (**d**) c/a=0.4, (**e**) c/a=0.5, (**f**) c/a=0.6, (**g**) c/a=0.7, and (**h**) c/a=0.8. For each c/a ratio, an inset illustrates the corresponding metamaterial unit cell.

The choice of the structure parameters shown in Figure 1 and used in Figure 2 as well as in the experiments to be described in the following section has resulted from a trade-off between experimental practicability, magnitude of acoustical activity, and bandwidth of acoustical activity. To help the reader appreciating the complexity of the behavior, we show in Figure 3 a series of band structures for the bulk case of $N_{x}=N_{y}=N_{z}\;\to \;\infty$. Here, as usual, the twist bands are completely absent due to the three-dimensional Bloch-periodic boundary conditions. We fix the lattice constant a and vary the ratio c/a, i.e., we vary the size of the inner cube in Figure 1. All other parameters are as in Figure 1 and Figure 2. We depict values ranging from c/a=0.1 to c/a=0.8. While the behavior remains qualitatively the same when going from c/a=0.2 (identical to Figure 2) to c/a=0.1 in Figure 3, the red transverse bands develop into a bubble-like structure towards intermediate values of c/a=0.5. Here, the two red bands are nearly degenerate of up to wave numbers of $k_{z}/a\;\approx \;1$. This behavior means that little if any acoustical activity appears below a certain minimum frequency of 150 kHz. Together with an upper maximum frequency of the bands of about 210 kHz, acoustical activity is expected to appear only in a narrow frequency range in this case. For yet larger ratios towards c/a=0.8, the band structures become again similar to the cases of c/a=0.1 and 0.2.

On this basis, we have selected c/a=0.2 (cf. Figure 1 and Figure 2) for our experiments. While many other geometrical parameters such as the b/a and the d/a ratio are not critical, it should be mentioned that it is important that the individual wires in the sets of wire bundles are not unintentionally connected on the way from one small cubic connection element to the next. Such connection would substantially reduce the twist of these beams, and hence the aimed-at effect (not depicted). This aspect has led us to stay away from yet smaller c/a ratios.

## 3. Experiments

**Figure 4:**
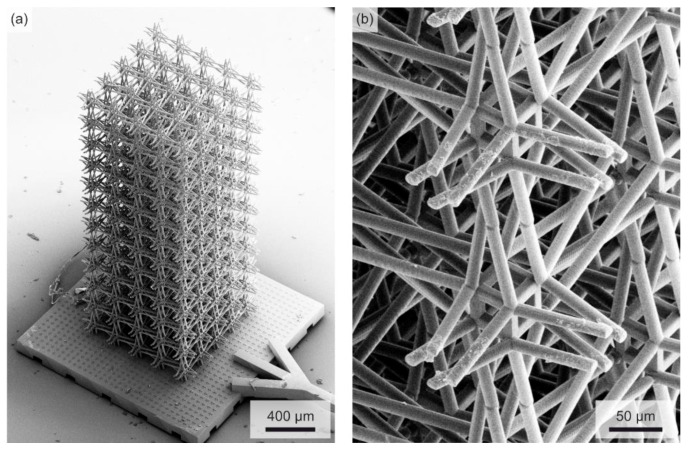
Selected oblique-view electron micrographs of a 3D chiral cubic polymer metamaterial sample manufactured by standard 3D laser micro-printing, following the blueprint illustrated in Figure 1. (**a**) Total view onto one metamaterial sample with $N_{x}\times N_{y}\times N_{z}=5\times 5\times 12$ unit cells and the bottom sample holder. Here, we use no plate at the top. (**b**) Zoom-in, showing the intricate interior composed of sets of twisted rods.

To test the above prediction of large polarization rotation angles (cf. Figure 2) for the architecture shown in Figure 1, we have manufactured corresponding polymer samples by standard 3D laser microprinting. The target parameters have been defined in Figure 1. Concerning the fabrication details, we refer the reader to Refs. [18,21] and the early work on “dip-in” mode [26], which is now widely used for the making of microstructured polymer-based 3D mechanical metamaterials by 3D laser nanoprinting [27]. We depict example electron micrographs in Figure 4. In contrast to our previous work [21], we have not added a plate at the sample top.

We sinusoidally excite the samples at their bottom by a piezoelectric actuator at frequency f=ω/(2π), with an amplitude of some 10 nm along the y-direction. We stroboscopically illuminate the samples by short pulses of two light-emitting diodes (850 nm center wavelength, 1.5% duty cycle), the repetition rate of which is synchronized with the piezoelectric excitation. We process the obtained microscope images at the top of the sample and at the sample bottom by using image cross-correlation analysis [18,21]. In this fashion, we can detect displacement vectors at different locations, $\vec{u}\left(\vec{r}\right)$, the moduli of which are much smaller than a pixel of the camera used for the recording and which are much smaller than the illuminating wavelength. At the sample bottom, we track the markers (cf. Figure 4); at the sample top, we track the ends of the four rods of the inner four unit cells, that is, 16 rod ends. By comparing the measured time-dependent displacement vectors at the sample bottom and sample top at a given frequency, we extract the polarization rotation angle φ, that is, the strength of acoustical activity. We refer interested readers to a more detailed description of this measurement setup given in Ref. [21].

## 4. Results and Discussion

**Figure 5:**
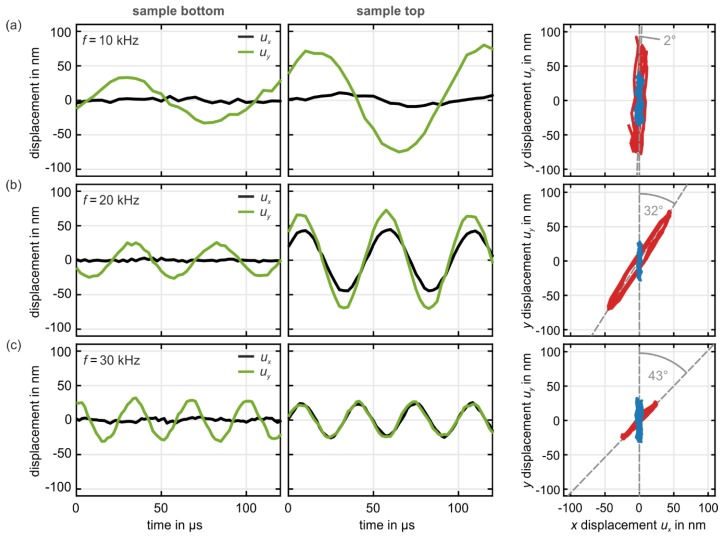
Measured displacement-vector components (black and green) versus time, taken at the sample bottom (left column) and at the top of the sample (middle column), respectively for three different frequencies f. The right column shows the y-component versus the x -component for the bottom (blue) as well as for the top (red). The sample is excited at its bottom by a piezoelectric transducer with (**a**) f=10 kHz, (**b**) f=20 kHz, and (**c**) f=30 kHz. The metamaterial beam has a cross section of $N_{x}\times N_{y}=3\times 3$ unit cells and a height of $N_{z}=12$ unit cells. From these example data, we derive a polarization rotation angle of (**a**) φ=2°, (**b**) φ=32°, and (**c**) φ=43°.

Example raw data for three selected excitation frequencies f are depicted in Figure 5. Here, $N_{x}=N_{y}=3$ and $N_{z}=12$. We show the individual x- and y-components of the displacement vector $\vec{u}=\left(u_{x},u_{y}\right)$ versus time at the sample bottom (left column) as well as at the sample top (middle column).

The right column shows the same data represented as y-component versus x-component for the sample bottom (blue) and the sample top (red). The extracted rotation angles φ are indicated, too.

**Figure 6:**
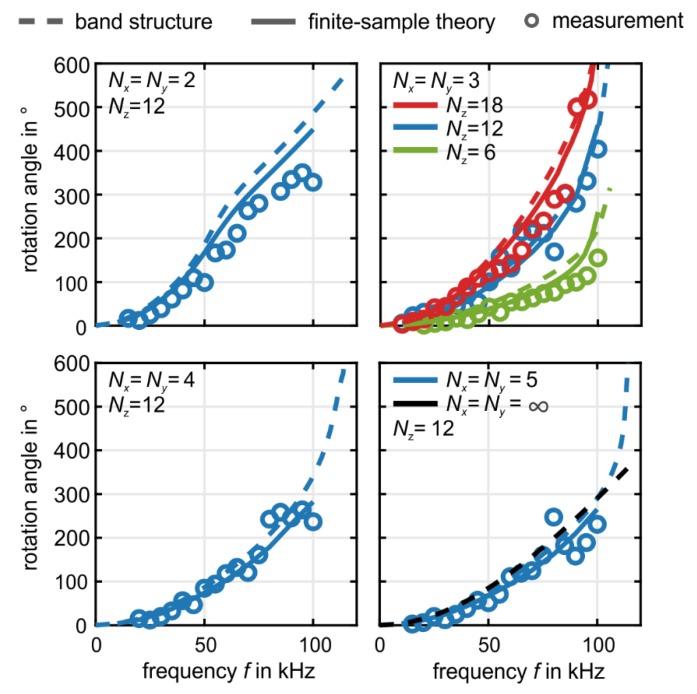
Derived from data like those shown in Figure 5, we plot the rotation angle φ=φ(f) versus excitation frequency f for different beam cross sections, $\left(N_{x}\times N_{y}\right)a^{2}$, and for different numbers of unit cells, $N_{z}$, along the propagation direction. The measured results are depicted as circles; the solid curve has been obtained from numerical finite-element frequency-domain calculations for finite-size samples (as in the experiment, cf. Figure 5), accounting for a finite imaginary part of the Young’s modulus E (cf. Figure 1); and the dashed curves have been obtained from phonon band-structure calculations (cf. Figure 2), assuming zero imaginary part of E. In the upper right-hand side panel, we vary $N_{z}$ at fixed $N_{x}=N_{y}$. In the lower right-hand side panel, we show by the dashed black curve the expectation for the bulk limit, obtained from phonon band-structure calculations, again with zero imaginary part of E. Obviously, the $N_{x}\times N_{y}=5\times 5$ case (blue) is already very close to the bulk limit (dashed back curve). At around f=100 kHz, we obtain a polarization rotation as large as about 30° per lattice constant.

A summary of many experiments (open dots) similar to the one shown in Figure 5, plotted in the form of rotation angle φ versus frequency f, with $N_{x}=N_{y}$ and $N_{z}$ as parameters, is depicted in Figure 6. The experiments are compared with numerical results from metamaterial-phonon band-structure calculations (dashed curves) and with numerical results for the finite samples as in the experiment. The finite length of the structure unavoidably leads to reflections. However, due to time-reversal symmetry, the rotation of the polarization is reversed on its way back and has the same direction as the excitation when it reaches the bottom of the sample again [21]. Therefore, the finite length of the sample influences the amplitude of the measured wave, yet it leaves the polarization direction unaffected [21]. 

We find the largest rotation angle per lattice constant of 30° for $N_{x}=N_{y}=2$. at f=100 kHz. The rotation angle generally decreases with increasing $N_{x}=N_{y}$. The 3D bulk limit ($N_{x}=N_{y}\;\to \;\infty$) is already approached at around $N_{x}=N_{y}=5$, for which we obtain about 20° polarization rotation angle per lattice constant at a frequency of f=100 kHz. The bulk limit of L/a → ∞ leads to a finite value of φ because the ratio of wavelength to lattice constant, λ/a, remains finite. Cauchy elasticity, for which the rotation effect would be zero, requires both, L/a → ∞ and λ/a → ∞, i.e., the large-sample limit and the static limit.

## 5. Conclusions

After briefly reviewing the history and the perspectives of the field of 3D mechanical metamaterials, we have presented our original results on the design, calculation of phonon band structures, manufacturing by 3D laser microprinting, and ultrasound experimental characterization of one type of chiral 3D mechanical metamaterial exhibiting acoustical activity—the mechanical counterpart of optical activity. Compared to our recent early corresponding results on a different kind of chiral cubic 3D micro-lattice, we have found enhanced effects in terms of the rotation angle per lattice constant in the ultrasound frequency range 10–100 kHz. The largest values found are around 45° rotation per lattice constant, in good agreement between theory and experiment. Notably, these values approach the fundamental bound of 90° rotation per lattice constant. Furthermore, the observed rotation angles decrease more slowly with increasing number of unit cells towards the bulk limit compared to our previous results. However, this advance comes at the price of a decreased robustness against fabrication errors, especially concerning the elongated twisted nearby yet non-touching rods, which form the heart of this chiral metamaterial. 

By decreasing the cubic lattice constant from the value of a=250 µm considered here, our results can be scaled to lower or larger operation frequencies. This possibility of scaling is a major advantage of chiral metamaterials compared to chiral ordinary crystals. For ordinary crystals, the maximum effects are much smaller to begin with. More importantly, they appear at a certain frequency (typically on the order of some THz), which is given by nature and which cannot be changed. Therefore, sizable effects of acoustical activity are not available from natural crystals at kHz and MHz frequencies, whereas they are available from 3D chiral mechanical metamaterials. Thereby, these metamaterials provide new degrees of freedom to control the polarization of elastic waves. This enables applications such as mode conversion from one incident transverse propagation to the orthogonal one.
